# Prevalence and Determinants of Academic Bullying Among Junior Doctors in Sierra Leone: Cross-Sectional Study

**DOI:** 10.2196/68865

**Published:** 2025-05-22

**Authors:** Fatima Jalloh, Ahmed Tejan Bah, Alieu Kanu, Mohamed Jan Jalloh, Kehinde Agboola, Monalisa M J Faulkner, Foray Mohamed Foray, Onome T Abiri, Arthur Sillah, Aiah Lebbie, Mohamed B Jalloh

**Affiliations:** 1 College of Medicine and Allied Health Sciences, University of Sierra Leone, Freetown, Sierra Leone; 2 Department of Public Health, Chamberlain College of Health Professions, Chicago, IL, United States; 3 University of Sierra Leone Teaching Hospitals Complex, Freetown, Sierra Leone; 4 College of Health Sciences and Public Policy, Walden University, Minneapolis, MN, United States; 5 School of Public Health, University of Washington, Seattle, WA, United States; 6 Faculty of Health Sciences, Department of Medicine, McMaster University, 1280 Main Street West, Hamilton, ON, L8S 4L8, Canada, 1 9059622812

**Keywords:** academic bullying, junior doctors, Sierra Leone, mental health, professional development

## Abstract

**Background:**

Academic bullying among junior doctors—characterized by repeated actions that undermine confidence, reputation, and career progression—is associated with adverse consequences for mental health and professional development.

**Objective:**

This study aimed to investigate the prevalence and determinants of academic bullying among junior doctors in Sierra Leone.

**Methods:**

We conducted a cross-sectional survey of 126 junior doctors at the University of Sierra Leone Teaching Hospitals Complex in Freetown between January 1 and March 30, 2024. Participants were selected through random sampling. Data were collected using a semistructured, self-administered questionnaire and analyzed with descriptive statistics and multivariable logistic regression.

**Results:**

Of the 126 participants (n=77, 61.1% male; mean age 31.9, SD 5.05 years), 86 (68.3%) participants reported experiencing academic bullying. Among those, 55.8% (n=48) of participants experienced it occasionally and 36% (n=31) of participants experienced it very frequently. The most common forms were unfair criticism (n=63, 73.3%), verbal aggression (n=57, 66.3%), and derogatory remarks (n=41, 47.7%). Consultants and senior doctors were the main perpetrators, with incidents primarily occurring during ward rounds, clinical meetings, and academic seminars. No statistically significant predictors of bullying were found for gender (odds ratio 2.07, 95% CI 0.92‐4.64; *P*=.08) or less than 2 years of practice (odds ratio 0.30, 95% CI 0.05‐1.79; *P*=.19).

**Conclusions:**

Academic bullying is widespread among junior doctors at the University of Sierra Leone Teaching Hospitals Complex. It has serious consequences for their mental health and professional development. There is an urgent need for clear and culturally appropriate policies, targeted training programs, confidential reporting systems, and leadership development. Promoting ethical leadership and fostering a culture of respect can help reduce incivility and burnout, leading to a healthier work environment for junior doctors.

## Introduction

Academic bullying—defined as maltreatment within academic settings intended to hinder the professional or academic progress of targeted individuals—remains a pervasive issue in medicine, particularly affecting junior doctors [1]. The hierarchical and high-stress nature of the medical profession, coupled with cultural norms that prioritize deference to authority, often creates environments in which public humiliation, verbal abuse, micromanagement, excessive workloads, and exclusion can flourish without adequate recourse [1-3]. Such repeated behaviors not only undermine the mental health and career development of junior doctors but also disrupt professional interactions and teamwork, potentially threatening patient safety and compromising the broader health care system [4].

Extensive research in high-income countries has consistently documented the widespread nature of bullying among junior doctors [1,4-6]. However, data from low-resource settings remain scarce. This paucity of information is especially concerning in Sierra Leone, where health care institutions grapple with significant resource limitations, workforce shortages, and constrained opportunities for professional development [7,8]. In these contexts, academic bullying may further intensify existing challenges, contributing to poor morale, reduced retention, and impaired patient care.

Adding to the urgency of investigating bullying within Sierra Leone’s health care sector are studies that have already documented alarmingly high rates of bullying in the country’s educational system. Research among in-school adolescents found a bullying prevalence of 48.7%, driven by factors such as loneliness, substance use, and school truancy [9]. School-related gender-based violence reports also confirm pervasive verbal and physical bullying, exacerbated by entrenched sociocultural norms and insufficient reporting mechanisms [10]. Although these data are drawn from younger populations, the same power imbalances and cultural drivers of bullying likely persist in higher education and professional settings. Indeed, the limited infrastructure for reporting and addressing maltreatment may allow such behaviors to continue into advanced academic and clinical environments.

Junior doctors in Sierra Leone are especially vulnerable to academic bullying due to strict hierarchies and limited resources, which can worsen their impact on both their well-being and the health care system. Despite the need for effective interventions, there is a lack of empirical data on the prevalence, determinants, and consequences of academic bullying among this demographic. Therefore, this study aims to investigate the prevalence of academic bullying among junior doctors at the University of Sierra Leone Teaching Hospitals Complex (USLTHC) in Freetown, Sierra Leone, and to examine the factors contributing to these behaviors. By situating the research within broader educational challenges and drawing on insights from prior studies, we seek to inform strategies for creating safer, more inclusive environments that support both the professional growth of junior doctors and the effective delivery of health care services.

## Methods

### Study Design and Setting

We conducted a cross-sectional survey at the major hospitals of USLTHC in Freetown, Sierra Leone. The USLTHC - Connaught Hospital, Princess Christian Maternity Hospital, Ola During Children’s Hospital, and Sierra Leone Psychiatry Teaching Hospital are the largest and primary government referral hospitals in the country and serve as the main training centers for junior doctors, including registrars (residents) and house officers (interns). In Sierra Leone, the term “junior doctor” refers to physicians who have not yet achieved full specialist (consultant) status. This includes those in postgraduate training or supervised practice, such as house officers (interns), who are recent medical graduates undergoing closely supervised practice; medical officers, who have completed internships and can work more independently but have not pursued formal residency training; and registrars (residents), who are enrolled in specialty training programs but have not yet attained full accreditation as specialists. The survey was conducted from January 1, 2024, to March 30, 2024.

### Participants and Sampling

All junior doctors who had been employed for a period of 6 months or longer and had reached the age of 18 years or older were included in the study. Those who were on outside posting or leave (annual or sick) were excluded, and no visiting junior doctors outside of USLTHC were included. The 6-month working experience requirement was used as the cutoff to ensure that participants have had sufficient interaction with both superiors and contemporaries during their training or postings.

### Sampling Strategy and Sample Size

We constructed our sampling frame by compiling a list of all junior doctors aged 18 years or older who had been employed at the USLTHC for at least 6 months. From this roster, we used a computer-based random selection procedure (ie, assigning unique identifiers and using a random number generator) to ensure that each eligible junior doctor had an equal probability of inclusion. This method was chosen to maintain methodological rigor despite the logistical challenges posed by frequent 3- to 6-month rotations.

To determine the sample size for the study, we used the Yamane formula for cross-sectional studies: n=*N*/(1+ N[e^2^]), where n is the required sample size, *N* is the total population size, and e is the margin of error set at 5% (0.05) [11].

Based on an estimated population of 160 eligible junior doctors, we calculated a minimum sample size of 114. Anticipating potential nonresponse or incomplete data, we increased this figure by 10% to arrive at a final target of 126 participants. Selected participants were drawn from multiple departments across four sites of the USLTHC.

### Data Collection Instrument

Data were collected using a semistructured, self-administered questionnaire, also offered via web (via a secure server using Microsoft Forms) for participants who could not complete the paper-based version. The survey captured demographic details (eg, sex, age, duration of practice or training, and job title) and focused on first-hand encounters with workplace bullying within the preceding 6 months. Participants who reported bullying were asked to describe these incidents, ensuring the data represented direct, personal experiences rather than observations of others being bullied.

The primary outcome measure was the respondent’s experience of workplace bullying, determined by a yes or no response to the question: “Have you experienced any form of workplace bullying in the last six months while training?”

Bullying was defined as repeated behaviors involving intimidation, humiliation, degradation, misuse of power, or abuse of authority that made the individual feel defenseless and undermined their dignity [1,2,12]. This definition guided our questionnaire design to differentiate self-reported experiences as a survivor from witnessing such acts. Before the main data collection, the survey instrument was piloted with 10 participants to confirm clarity and relevance, with refinements made based on their feedback.

### Statistical Analysis

We conducted descriptive statistics to summarize the data. For continuous variables with a normal distribution, we reported means and SDs; for nonnormally distributed variables, medians and IQRs were provided. Associations between categorical variables were assessed using Pearson χ^2^ tests or Fisher exact tests, as appropriate. Results were presented in tables and graphical summaries.

To explore independent associations between prespecified characteristics and the primary outcome—respondents’ experience of workplace bullying—we performed multivariable logistic regression analyses. Explanatory variables were selected based on their relevance and included age (≤34 y vs ≥35 y), sex (male vs female), marital status (married vs others), level of training (house officer and others vs registrar), and duration of practice (≤2 y vs ≥3 y). Results were reported as odds ratios (ORs) with 95% CIs and corresponding *P* values. Statistical significance was set at a 5% level. All analyses were conducted using SPSS (version 27; IBM Corp).

### Participant and Public Involvement Statement

Due to unexpected delays and time constraints, we were unable to involve participants or the public in the study’s design, execution, or reporting. However, we are now considering a higher level of public and stakeholder engagement when sharing our research findings.

### Ethical Considerations

The study received ethics approval from the College of Medicine and Allied Health Sciences Institutional Review Board (review number: COMAHS/IRB/013‐2024). All procedures involving human participants were conducted in accordance with the ethical standards of the institutional and national research committee and with the 1964 Declaration of Helsinki and its later amendments. Informed consent was obtained from all participants prior to their completion of the questionnaire. Participation was voluntary, and participants were informed about the purpose of the study, their right to withdraw at any time, and the measures in place to protect their data. No compensation was provided to participants for their involvement in this study. All responses were collected anonymously, and no personally identifiable information was obtained. Strict confidentiality protocols were followed, including secure data storage and restricted access, to ensure the privacy and integrity of the data.

## Results

### Sociodemographic Characteristics of Participants

A total of 126 individuals completed the survey, comprising 77 (61.1%) male and 49 (38.9%) female participants. The mean age of the participants was 31.9 (SD 5.05) years. Regarding marital status, 68 (53.9%) individuals were single and never married, 52 (41.3%) individuals were married or in a domestic partnership, 2 (1.6%) individuals were separated, 1 (0.8%) individual was divorced, and 3 (2.4%) individuals preferred not to disclose their marital status (Table 1).

**Table 1.** Sociodemographic characteristics of the respondents (n=126).

Characteristics	Frequency
Age (years), n (%)	
18‐24	2 (1.6)
25‐34	95 (75.4)
35‐44	26 (20.6)
45‐54	3 (2.4)
Age (years), mean (SD)	31.9 (5.05)
Sex, n (%)	
Female	49 (38.9)
Male	77 (61.1)
Marital status, n (%)	
Single, never married	68 (53.9)
Married or domestic partnership	52 (41.3)
Separated	2 (1.6)
Divorced	1 (0.8)
Prefer not to say	3 (2.4)
Level of training, n (%)	
House officer	59 (46.8)
Medical officer	22 (17.5)
Registrar	43 (34.1)
Senior registrar	2 (1.6)
Duration of practice (years), n (%)	
<2	66 (52.4)
2 and above	60 (47.6)
Current training department, n (%)	
Internal medicine	35 (27.8)
Surgery and its subspecialties	34 (26.9)
Pediatrics	21 (16.7)
Obstetrics and gynecology	23 (18.3)
Family medicine	5 (3.9)
Psychiatry	6 (4.8)
Laboratory medicine	2 (1.6)

In terms of level of training, the sample included 59 (46.8%) house officers, 22 (17.5%) medical officers, 43 (34.1%) registrars, and 2 (1.6%) senior registrars. The duration of practice varied, with 66 (52.4%) participants having practiced for 2 years or less and 60 (47.6%) participants having practiced for 3 years or more (Table 1).

Participants were also categorized by their current training departments. Internal medicine had the highest representation, with 35 (27.8%) individuals, followed by surgery and its subspecialties with 34 (26.9%) individuals. Pediatrics included 21 (16.7%) participants, obstetrics and gynecology had 23 (18.3%) participants, family medicine included 5 (3.9%) participants, psychiatry had 6 (4.8%) participants, and laboratory medicine included 2 (1.6%) participants (Table 1).

This study examined the prevalence and forms of academic bullying among 126 participants. A total of 86 (68.3%) individuals reported experiencing bullying, while 40 (31.8%) individuals did not report such experiences (Table 2). Among the participants who reported being bullied, 48 (55.8%) experienced bullying occasionally, and more than one-third (36%) experienced bullying very frequently (Table 3).

**Table 2.** Prevalence and forms of academic bullying.

Variable	Frequency, n (%)
Current experience of bullying (n=126)	
Experienced	86 (68.3)
Not experienced	40 (31.8)
Forms of bullying (n=86)^a^	
Unfair criticism or evaluation	63 (73.3)
Verbal aggression	57 (66.3)
Derogatory remarks	41 (47.7)
Threat or intimidation	33 (38.4)
Undermining dignity at work	30 (34.9)
Exclusion from academic activities	16 (18.6)
Others (extra on-call service)	1 (1.2)
Common perpetrators of bullying (n=86)	
Consultants	72 (83.7)
Other senior doctors (colleagues)	66 (76.7)
Nursing staff	18 (20.9)
Administrative staff	15 (17.4)
Peers	13 (15.1)

a Percentages are calculated based on the total number of respondents who reported any form of bullying or reported any type of perpetrator (n=86).

**Table 3.** Frequency of bullying experienced by junior doctors.

Bullying frequency	Frequency, n (%)
Occasionally	48 (55.8)
Very frequently	31 (36.0)
Rarely	6 (7.0)
Always	3 (3.5)

Among those who reported experiencing bullying (n=86), the most common forms of bullying included unfair criticism or evaluation, reported by 63 (73.3%) individuals, and verbal aggression, reported by 57 (66.3%) individuals. Derogatory remarks were reported by 41 (47.7%) individuals, and threats or intimidation were experienced by 33 (38.4%) individuals. Other reported forms of bullying included undermining dignity at work (30/86 individuals, 34.9%), exclusion from academic activities (16/86 individuals, 18.6%), and extra on-call service demands (1/86 individuals, 1.2%) (Table 2).

Regarding the common perpetrators of bullying (n=86), consultants were identified as the most frequent perpetrators, reported by 72 (83.7%) individuals. Other senior doctors were reported by 66 (76.7%) individuals as perpetrators. Additionally, 18 (20.9%) individuals reported nursing staff as perpetrators, 15 (17.4%) individuals reported administrative staff, and 13 (15.1%) individuals reported peers as perpetrators of bullying (Table 2).

The most common context or setting in which academic bullying occurred was during ward rounds, reported by 73 (84.9%) participants. Clinical meetings were another context in which 51 (59.3%) individuals experienced bullying. A total of 50 (58.1%) individuals reported academic seminars or presentations as the context for bullying. Last, administrative meetings were identified as a bullying setting by 8 (9.3%) individuals (Table 4).

**Table 4.** Context or setting of bullying activity.

Context/setting	Frequency, n (%)
During ward rounds	73 (84.9)
Clinical meetings	51 (59.3)
Academic seminars or presentations	50 (58.1)
Administrative meetings	8 (9.3)

### Multiple Logistic Regression Analysis of Factors Independently Associated With Bullying

The logistic regression analysis did not identify any statistically significant predictors of bullying at the 5% significance level. Participants aged 35 years or older had 0.78 times the odds of experiencing bullying compared with those aged 34 years or younger (OR 0.78, 95% CI 0.29-2.14; *P*=.63). House officers had 0.66 times the odds of experiencing bullying compared with registrars (OR 0.66, 95% CI 0.10-4.34; *P*=.67), while participants in the “Others” designation category (medical officers and senior registrars) had 2.58 times the odds of experiencing bullying compared with registrars (OR 2.58, 95% CI 0.67-9.92; *P*=.17). Marital status showed that participants categorized as “Others” had 0.94 times the odds of experiencing bullying compared with married or domestic partnership participants (OR 0.94, 95% CI 0.38-2.35; *P*=.90) (Table 5).

**Table 5.** Multiple logistic regression analysis of factors independently associated with bullying.

Factors	OR^a^ (95% CI)	*P* value
Sex		
Female^b^	1	—^c^
Male	2.07 (0.92‐4.64)	.08
Age (years)		
≤34^b^	1	—
35 or older	0.78 (0.29‐2.14)	.63
Marital status		
Married or domestic partnership^b^	1	—
Others	0.94 (0.38‐2.35)	.90
Level of training		
Registrar^b^	1	—
House officer	0.66 (0.10‐4.34)	.67
Others	2.58 (0.67‐9.92)	.17
Duration of practice (years)		
2 or more^b^	1	—
<2	0.30 (0.05‐1.79)	.19
Intercept	3.00 (0.38‐23.45)	.29

a OR: odds ratio.

b Reference categories that serve as the baseline for comparison.

c Not applicable.

Male participants had 2.07 times the odds of experiencing bullying compared with female participants (OR 2.07, 95% CI 0.92-4.64; *P*=.08). Participants with <2 years of practice had 0.30 times the odds of experiencing bullying compared with those with more than 2 years of practice (OR 0.30, 95% CI 0.05-1.79; *P*=.19) (Table 5).

The intercept, representing the log odds of experiencing bullying for the reference category (≤34 years old, female, married, registrar, ≥3 years of practice), had an OR of 3.00 (95% CI 0.38-23.45; *P*=.29), which serves as the baseline for comparison but is not directly interpretable in the same way as the other predictors (Table 5).

## Discussion

### Principal Findings

In this cross-sectional study, we investigated the prevalence and determinants of academic bullying among junior doctors at USLTHC in Freetown, Sierra Leone, between January 1 and March 30, 2024. We found a high prevalence of bullying (68.3%) among 126 participants, with unfair criticism and verbal aggression being the most common forms. Consultants and other senior doctors were frequently identified as perpetrators. Bullying occurred most frequently during ward rounds and clinical meetings. Despite the high prevalence, the analysis did not find any factors that were significantly associated with the likelihood of experiencing bullying.

The high prevalence of academic bullying in this study is much higher than the global average reported in systematic reviews, which found an overall prevalence of 51% (95% CI 36%‐66%) [4]. However, this finding aligns more closely with data from sub-Saharan Africa, exceeding the prevalence reported in Nigeria (59.7%) [2] but lower than that in Ghana (82%) [13]. These results suggest that while the prevalence of academic bullying in our study surpasses the global norm, it is consistent with regional trends.

Bullying predominantly occurred during ward rounds (84.9%), clinical meetings (59.3%), and academic seminars (58.1%), consistent with literature indicating that hierarchical settings in medical environments are common contexts for such behavior [14,15]. Multiple forms of bullying were identified, including unfair criticism, verbal aggression, derogatory remarks, and threats or intimidation. Consultants were the most frequently reported perpetrators, aligning with findings from a systematic review where 53.6% of 15,868 respondents identified senior staff as bullies [1]. These observations underscore the influence of entrenched power dynamics within the medical profession on bullying behaviors [16].

The high prevalence of bullying in our sample population can be attributed to several factors inherent in the medical profession. Hierarchical power dynamics, overwhelming workloads, and a lack of institutional support have been noted in other studies and are evident in our setting [14]. Bullying often occurs hierarchically, with senior staff perpetrating negative behaviors toward junior colleagues [15]. The Joint Commission has emphasized that health care professionals in positions of power commonly exhibit intimidating and disruptive behaviors, highlighting the systemic nature of the issue [16].

Toxic work cultures—including bullying and discrimination—are significant sources of distress for junior doctors, necessitating urgent institutional interventions. In Sierra Leone, medical professionals face escalating demands, diminishing resources, and staff shortages, factors known to compound psychological distress [7]. These stressors not only increase the risk of being bullied but also exacerbate the situations under which bullying occurs and intensify its negative impact. The absence of structured systems to counteract this culture may explain the high prevalence observed. Further research is needed to elucidate the role of these stressors, specifically related to perpetrators of bullying in the medical profession.

### Determinants of Bullying in the Medical Profession

Our study found no significant differences in the incidence of bullying across demographic factors such as gender, age, marital status, designation, or duration of practice. While previous studies suggest a higher incidence of bullying against females [1,5]—and considering the patriarchal context of Sierra Leone—our data did not reflect significant gender differences. This may be due to reporting biases or specific workplace dynamics and aligns with findings from similar studies in the subregion [13,17]. These results underscore the need for further research and qualitative exploration to uncover underlying factors contributing to bullying.

Similarly, our findings deviate from other studies reporting higher odds of bullying among younger and less experienced individuals, attributed to lower status, perceived vulnerability, and power dynamics [18]. Studies have shown that individuals who are separated, divorced, or widowed have higher odds of reporting bullying than married individuals [19]. However, our study found no statistically significant correlation between marital status and reports of bullying.

The lack of statistically significant findings may be due to sample homogeneity; a more extensive and diverse sample could provide greater insight into demographic determinants of bullying, highlighting the need for further studies. Given the homogeneity of our sample, exploration of factors such as race-related bullying, which has been shown to lead to profound psychological distress, was not applicable [5].

### Impact of Academic Bullying in the Medical Profession

Academic bullying has profound impacts on the medical profession. The hierarchical nature of medical training can lead to burnout and dissatisfaction among medical students and residents, deterring them from pursuing further specialization or academic careers [20]. This underscores the broader influence of workplace dynamics on health care professionals’ career trajectories and well-being. In Sierra Leone, already facing a shortage of specialized medical staff, the negative effects of academic bullying may exacerbate this issue [7]. Research has demonstrated that victims of bullying may become perpetrators themselves, perpetuating a cycle particularly evident in hierarchical structures where each level may bully the one below [21].

Studies have highlighted the psychological impact of workplace bullying on junior doctors, including its associations with common mental disorders and suicidal ideation. The detrimental effects extend beyond direct victims to colleagues who may be vicariously impacted. Organizational factors, such as climate, culture, leadership, and support, play significant roles in predicting exposure to bullying, emphasizing the need for holistic approaches to address workplace victimization.

Research has also explored the relationship between workplace bullying and employee turnover intentions, as well as negative implications for productivity and teamwork [22]. The psychological and emotional distress caused by bullying affects both the personal and professional lives of junior doctors [23], a critical concern for nations like Sierra Leone grappling with medical professional shortages. While coping mechanisms such as seeking peer support and focusing on personal growth are used [24], systemic changes are imperative to address the root causes of bullying in academic settings. Recognizing workplace bullying as a systemic problem necessitates comprehensive solutions to foster a more supportive and respectful work environment.

### Practical Implications

To effectively address academic bullying within USLTHC and the broader Sierra Leone health care system, a comprehensive, evidence-based approach is necessary. Establishing culturally sensitive antibullying policies is imperative to create a safer and more respectful academic environment. Implementing comprehensive training programs for medical staff—focused on recognizing and preventing bullying, promoting respectful communication, and fostering supportive work environments—is essential. Moreover, advocating for authentic leadership that empowers junior doctors, promotes transparent communication, and addresses hierarchical imbalances can substantially contribute to the mitigation of bullying behaviors in health care settings [25].

Confidential reporting channels, such as anonymous hotlines or independent web-based platforms, are vital for safeguarding individuals and promoting whistleblowing. Enhancing leadership development within the medical hierarchy is also crucial. Effective leadership models in health care enhance learning, teaching, and patient care. By fostering ethical leadership principles, health care organizations can cultivate a culture of respect, integrity, and accountability [26].

Ethical leadership profoundly influences health care outcomes, including job satisfaction, safety compliance, and reduction of workplace deviance. The positive impact of ethical leadership on job satisfaction enhances service quality, patient satisfaction, and productivity [27]. Ethical leadership improves safety compliance by building trust among health care professionals [28]. Fostering a culture of trust and ethical behavior is therefore crucial for promoting positive outcomes in health care organizations.

Addressing incivility and unethical behaviors in health care settings is essential. Organizations can leverage Ethics Committees and Clinical Ethics Consultation Services to manage incivility and promote ethical practices [29]. Integrating ethical considerations into organizational practices fosters a supportive and respectful work environment, aligning with the need to cultivate ethical leadership skills among health care professionals [30].

Implementing antibullying interventions and creating supportive environments through mentorship, coaching, and feedback mechanisms can mitigate the negative impacts of bullying on junior doctors [31,32]. Fostering a culture of respect and support within medical institutions is essential to promoting the well-being and professional development of all health care professionals, including junior doctors [20,33].

### Strengths and Limitations

This study represents the first investigation into academic bullying among junior doctors in Sierra Leone. Strengths include the straightforward administration of the survey, facilitated by a well-educated study population and a readily accessible participant list.

However, several limitations must be acknowledged. The reliance on self-reported experiences introduces the potential for response bias, including underreporting due to fear of administrative scrutiny. Additionally, there is a lack of a validated instrument for evaluating academic bullying in an African context. The questionnaire was developed based on prior studies and an extensive literature review. Despite these constraints, the findings suggest disturbingly high levels of perceived bullying and mistreatment during training. Results should be interpreted cautiously, and a higher response rate would have been preferable.

### Conclusions

This study revealed a high prevalence of academic bullying among junior doctors at USLTHC, with unfair criticism, verbal aggression, derogatory remarks, and threats or intimidation being the most common forms identified. Consultants and other senior doctors were frequently identified as perpetrators. Bullying most commonly occurs during ward rounds and clinical meetings. Despite the high prevalence, the analysis did not find any sociodemographic factors significantly associated with the likelihood of experiencing bullying.

Academic bullying in medicine undermines junior doctors’ mental health and professional development, compromising both individual well-being and the quality of patient care. Confronting this pervasive issue within USLTHC and the broader Sierra Leone health care system demands a comprehensive, evidence-based strategy.

## Abbreviations

OR: odds ratio
USLTHC: University of Sierra Leone Teaching Hospitals Complex
